# Housing Instability Associated with Return to Stimulant Use among Previously Abstaining Women

**DOI:** 10.3390/ijerph20196830

**Published:** 2023-09-26

**Authors:** Nicky J. Mehtani, Chika C. Chuku, Meredith C. Meacham, Eric Vittinghoff, Samantha E. Dilworth, Elise D. Riley

**Affiliations:** 1 Department of Psychiatry & Behavioral Sciences, University of California, San Francisco, CA 94143, USA; meredith.meacham@ucsf.edu; 2 Whole Person Integrated Care, San Francisco Department of Public Health, San Francisco, CA 94103, USA; 3 Department of Public Health Sciences, University of Miami, Coral Gables, FL 33136, USA; ccc162@miami.edu; 4 Department of Epidemiology and Biostatistics, University of California, San Francisco, CA 94143, USA; eric.vittinghoff@ucsf.edu; 5 Department of Medicine, University of California, San Francisco, CA 94143, USA; samantha.dilworth@ucsf.edu (S.E.D.);

**Keywords:** homelessness, housing instability, women, methamphetamine, cocaine, housing policy, stimulant use disorder

## Abstract

Stimulant use among unstably housed individuals is associated with increased risks of psychiatric co-morbidity, violence, HIV transmission, and overdose. Due to a lack of highly effective treatments, evidence-based policies targeting the prevention of stimulant use disorder are of critical importance. However, little empirical evidence exists on risks associated with initiating or returning to stimulant use among at-risk populations. In a longitudinal cohort of unstably housed women in San Francisco (2016–2019), self-reported data on stimulant use, housing status, and mental health were collected monthly for up to 6 months, and factors associated with initiating stimulants after a period of non-use were identified through logistic regression. Among 245 participants, 42 (17.1%) started using cocaine and 46 (18.8%) started using methamphetamine. In analyses adjusting for demographics and socio-structural exposures over the preceding month, experiencing street homelessness was associated with initiating cocaine use (AOR: 2.10; 95% CI: 1.04, 4.25) and sheltered homelessness with initiating methamphetamine use (AOR: 2.57; 95% CI: 1.37, 4.79). Other factors—including race, income, unmet subsistence needs, mental health, and treatment adherence—did not reach levels of significance, suggesting the paramount importance of policies directed toward improving access to permanent supportive housing to prevent stimulant use among unstably housed women.

## 1. Introduction

In recent years, the incidence of stimulant drug use has risen dramatically. In the United States, this has been marked by a 50-fold increase in the number of overdose deaths involving amphetamines and a 5.6-fold increase in those involving cocaine over the past two decades [1]. Consistently ranked among the most harmful substances both to individuals and society [2,3], methamphetamine and cocaine have cardio- and neurotoxic properties [4,5,6,7] that substantially increase individuals’ risks of experiencing physical and mental illness, including myocardial infarction, stroke, psychosis, and cognitive decline [8,9].

Increasing rates of stimulant use among people experiencing homelessness (PEH) and unstable housing are of particular concern due to the compounding nature of adverse socio-structural exposures faced by these vulnerable populations. The use of stimulants among unstably housed persons has been associated with increased use of other controlled substances, worsening mental health outcomes, HIV and HCV seroconversion, intimate partner violence, crime, incarceration, reduced adherence to treatments for medical co-morbidities, and premature death [10,11,12,13,14,15].

While methamphetamine and cocaine have considerable overlap with regard to risk factors and pathophysiologic effects, the two drugs have important differences in pharmacokinetics, routes of administration, costs, and user demographics, which vary across geographic locations [16,17,18,19,20]. In contrast to cocaine, which is metabolized by the body within 1–2 h, methamphetamine has a longer duration of action and a plasma half-life of 6–17 h. This results in methamphetamine remaining in the brain for longer than cocaine, leading to prolonged stimulant effects [21]. While cocaine prolongs dopamine’s actions by blocking the re-uptake of the neurotransmitter in the brain, methamphetamine not only blocks dopamine re-uptake but also increases the release of dopamine into the synaptic cleft between neurons—an effect that often results in the development of neuronal toxicity [21]. The two drugs also differ with regard to demographic and geographic predispositions among users. While patterns of drug use across the U.S. are constantly evolving and both drugs are prevalent in urban centers, historically, rates of methamphetamine use have been greatest in the West and Midwest, with decreased prevalence in many northeastern cities [22], where cocaine use has predominated. In San Francisco, 53% of drug overdose deaths in 2021 involved methamphetamine and 37% involved cocaine [23].

Notwithstanding a large body of research investigating potential therapeutic interventions, existing evidence-based treatments for stimulant use disorder have been sub-optimal in scope, accessibility, and/or efficacy [24,25,26]. Contingency management (CM)—a behavioral approach involving immediate, tangible rewards to reinforce positive behaviors—is currently the most effective treatment [26]. However, CM has generally lacked sustained efficacy following the termination of incentives and is difficult to administer in real-world settings due to resource limitations [27]. Several pharmacotherapies have also been studied but have shown minimal efficacy [24,25,28,29,30]. In practice, treatment for stimulant use disorder often consists of counseling alone, despite a lack of demonstrative efficacy and high treatment drop-out rates—estimated at 53.5% for methamphetamine and 48.7% for cocaine, higher than those for any other substance use disorders (SUDs) evaluated in a recent systematic review [31].

Given this lack of availability of effective pharmacologic and non-pharmacologic therapies for stimulant use disorder, public health policies aimed at preventing the onset of or the return to stimulant use among at-risk populations are of critical importance. However, few studies have identified specific prevention strategies or risk factors related to the initiation or re-initiation of stimulant use in disadvantaged populations. Even fewer have done so among very low-income women, which is a salient point given known sex-related differences in stimulant craving, addiction, and relapse [32,33].

The current analysis sought to measure the impact of socio-structural exposures that may be less common in the general population but are highly prevalent among impoverished individuals—including low-income status, homelessness, unmet subsistence needs, specific mental health conditions, and sub-optimal medication treatment adherence—on the likelihood of initiating or returning to methamphetamine or cocaine use in a cohort of unstably housed women. While our prior work suggests that experiencing homelessness or recent sexual violence increases women’s likelihood of initiating stimulants [12], the current study evaluated the relative impact of different types of homelessness (i.e., sleeping on the street vs. in a shelter; not being able to stay in the same place for more than two weeks; or choosing a location to sleep based on avoiding violence) separately for methamphetamine and cocaine using a recently sampled cohort. Given the increased apparent vulnerability among individuals experiencing street homelessness, we hypothesized that sleeping on the street or in a public place would be more strongly associated with stimulant drug initiation than other forms of homelessness. However, reports have suggested that temporary housing and other forms of sheltered homelessness are associated with experiences of violence and temporal uncertainty [34], which could also pre-dispose individuals to use stimulants. Findings from this analysis may inform the design and development of evidence-based policies and targeted interventions to help prevent the initiation of or return to stimulant use among vulnerable patients.

## 2. Materials and Methods

Study Population—This analysis utilized data from the PULSE (Polysubstance Use and Health Outcomes Evaluation) study, a longitudinal bio-behavioral study examining the impact of HIV and substance use on the cardiac health of unstably housed women between June 2016 and January 2019 [35]. Eligible participants included individuals who were born female, aged 18 or older, and had a lifetime history of sleeping on the street or in homeless shelters or staying with a series of acquaintances due to not having their own residence (i.e., “couch surfing”). Women were recruited from homeless shelters, all free meal programs serving over 100 meals per day, a probability sample of single room occupancy (SRO) hotels, an area sample of street encampments, and a public HIV clinic in San Francisco, CA, USA. At baseline, 31% of study participants had HIV, and 53% and 29% had toxicologically confirmed cocaine and methamphetamine use, respectively. Participants were reimbursed USD 40 per visit to attend six consecutive monthly study visits, each of which included a confidential interview, vital sign assessment, and blood draw.

Outcome—The primary dependent variable of interest was self-reported past-month use of methamphetamine or cocaine (including both powder cocaine and crack cocaine) after at least 1 study visit during which a participant reported having not used methamphetamine or cocaine. At each study visit, data on self-reported recent and lifetime use of methamphetamine and cocaine was collected via audio computer-assisted self-interview (ACASI). For each substance reported, participants were asked the time of last use, with options including the last week, month, year, and over a year ago.

Exposures—Independent variables included demographics at baseline as well as participant-reported exposures and behaviors over the preceding month, including housing conditions, mental health co-morbidities, and medication adherence. Sociodemographic characteristics included age, race, ethnicity, and income. We defined housing status based on HUD definitions of homelessness (§ 578.3) [36,37], including having slept at a homeless shelter or in a public place, choosing a place to sleep to avoid violence, or not being able to stay at the same place for the next two weeks. Additional variables included scores on validated measures of mental health, including for depression (PHQ-9), post-traumatic stress disorder (PTSD; (PCL)), anxiety (GAD-7), and cognitive function (PROMIS-8), as well as sexual orientation and adherence to treatment medications. Because prior studies have suggested that people who use drugs may substitute opioids or stimulants with marijuana to reduce perceived substance-related harms [38,39], using marijuana as a substitute for another substance was also included as a potential explanatory variable. Drug substitution questions were added in April 2018 and were only posed to participants who reported marijuana use. All other questions were asked throughout the study period and with all study participants.

Data Analysis—Multivariable logistic regression was used to estimate unadjusted and adjusted associations between the initiation of stimulant use after a period of non-use using two separate models, one for cocaine use and one for methamphetamine use. Variables that reached a level of significance in unadjusted analysis (*p* < 0.05) were included in multivariable models. Missing data were rare (<3%) and considered missing at random. Analyses were conducted in Stata Version 16.2 (Stat Corp., College Station, TX, USA).

## 3. Results

### 3.1. Participants

A total of 245 women were enrolled in the original study with a mean age of 51.6 years (SD, 10.8 years), including 74% of whom reported non-White race/ethnicity (Table 1). Across the cohort, 93 women (38%) reported experiencing homelessness—including 68 (27.8%) who had slept at a homeless shelter and 56 (22.9%) who had slept in a public place over the preceding month—and 87 (35.5%) reported having chosen a location to sleep based on avoiding violence. There was a significant burden of mental illness in the population, with 182 women (74.9%) confirming signs and symptoms consistent with depression, PTSD, or anxiety, 111 (61.0%) of whom were untreated or reported less than daily adherence to psychiatric medications.

### 3.2. Association between Study Factors and Stimulant Initiation

Forty-two women (17.1%) started using cocaine, forty-six (18.8%) started using methamphetamine, and four (2.2%) started using both after at least one study visit in which they had indicated not using these substances. Adjusted analyses demonstrated that the odds of starting to use cocaine after a period of non-use decreased by 42% for every 10 years of age (OR 0.58, 95% CI: 0.44, 0.78) and were twice as high among women who slept in a public place in the preceding month compared to those who did not sleep in a public place (OR 2.10, 95% CI: 1.04, 4.25) (Table 2). The odds of starting to use methamphetamine were 64% lower among women who had the ability to stay in the same residence for the next two weeks (OR 0.36, CI: 0.17, 0.79) and more than two and a half times as high among those who slept in a homeless shelter (OR 2.57, 95% CI: 1.37, 4.79) compared to those who did not (Table 3). Other known predictors of initiating stimulant use—including mental health diagnoses, income, unmet subsistence needs, and choosing a location to sleep based on avoiding sexual violence—did not reach levels of significance in this population.

## 4. Discussion

In this cohort of impoverished women living in a high-resource, densely populated urban setting, 17.1% started using cocaine and 18.8% started using methamphetamine after a period of non-use over 6 months of follow-up. Initiating cocaine use was associated with younger age and sleeping in a public place, while initiating methamphetamine was associated with sleeping in a homeless shelter and not being able to stay at one’s current residence for more than two weeks. Associations with other known risk factors, including race, income, unmet subsistence needs, mental illness, and treatment adherence, were not as strong in this analysis. These findings suggest that, among highly vulnerable women, experiencing homelessness—whether on the street or in homeless shelters—is a key modifiable risk factor for stimulant initiation, underscoring the critical importance of public health strategies directed toward improving population-level access to permanent supportive housing.

While few prior analyses have focused specifically on unstably housed women, these data corroborate findings from studies conducted with other high-risk patient populations linking substance use initiation or re-initiation with experiences of homelessness [12,13,40,41,42,43]. In alignment with the social causation hypothesis, which posits that people use substances in response to experiences of poverty-related trauma [44,45], qualitative reports have suggested that women use stimulants to cope with feelings of physical and emotional pain, including those imposed by structural or ‘everyday’ violence related to homelessness [46]. Living in unsafe environments may provoke feelings of anxiety and hypervigilance, which may trigger relapse or a compulsion to use stimulants in an attempt to escape the realities of social marginalization [47,48]. An ethnographic study also identified “functional” methamphetamine use related to a desire for increased alertness—enabling individuals to remain ‘on guard’ in the context of vulnerable living environments—as a key motivator for use among people experiencing homelessness (PEH) [49].

Findings from this analysis are also consistent with those from a recent systematic review which demonstrated a robust association between homelessness and the subsequent use of substances, though less conclusive findings regarding more broadly defined ‘housing stress’ and substance use [43]. While all participants in the current study had a history of unstable housing, of the risk factors measured, only experiences of distinct homelessness and currently active housing instability demonstrated robust associations with initiating methamphetamine or cocaine use. Thus, although “Housing First” approaches—in which the provision of housing is prioritized over addiction recovery—have demonstrated mixed results on substance use outcomes among PEH with SUDs [43], findings from this study strongly support policies that increase access to safe and permanent housing as a means of preventing the initiation of stimulant use by reducing vulnerability to experiences associated with street and sheltered homelessness.

By sampling participants from venues known to accommodate people with unmet subsistence needs and by restricting study enrollment to women with a history of housing instability, we adjusted a priori for social and structural factors known to influence substance use. Thus, the results reported here are specific to a highly vulnerable group and not the general population. The lack of association between mental health conditions and stimulant initiation may thus reflect a sample population with a substantial level of baseline mental illness and co-morbid SUDs [50,51,52,53,54]. For example, the results could suggest that study participants with pre-existing dual-diagnosed mental illness and SUDs had been using stimulants throughout the 6-month period of follow-up and, therefore, did not initiate or re-initiate stimulant use. The fact that specific types of homelessness stood out as being statistically significant in a cohort of more homogenously disadvantaged individuals than is usually represented in other research demonstrates the strong influence of housing on stimulant use in this population. It is also worth noting that, beyond literal homelessness, the imminent risk of homelessness (i.e., not being able to stay at one’s current residence beyond 14 days) was also associated with starting to use methamphetamine, further emphasizing the importance of stable housing to reduce rates of initiating or returning to stimulant use.

The findings that both street and sheltered homelessness were associated with stimulant use initiation are also notable and timely in the context of recent, high-profile advocacy efforts promoting the development of homeless shelters over permanent supportive housing in an attempt to address the concurrent drug and homelessness crises within U.S. cities [55,56]. This analysis, which demonstrated that sleeping in a homeless shelter was associated with methamphetamine use after a period of non-use, counters such arguments suggesting that homeless shelters be used to help people recover from drug use as a pre-requisite to housing eligibility for PEH. In contrast, the current study’s results lend credence to the idea that living at temporary locations such as homeless shelters results in the perpetuation of ‘temporal uncertainty’—defined as a painful and frustrating inability to move through time in desired ways due to housing instability [34]—thereby increasing one’s risk of using methamphetamine as a coping strategy.

The apparent associations between street homelessness with initiation of cocaine use and sheltered homelessness with methamphetamine use were unexpected and warrant further investigation. While this may potentially be attributable to un-measured confounders related to the specific characteristics of people who use cocaine vs. methamphetamine, the sample size in this study is too small to draw robust conclusions regarding the observed differences. However, this is an interesting finding that would benefit from further evaluation in future longitudinal and qualitative studies conducted with unstably housed women and people who use drugs. In addition, findings reported here suggest that prevention of homelessness may be key to reducing stimulant use among highly impoverished women, which is consistent with prior research showing that eviction predicts initiation or relapse into crystal methamphetamine use in mostly male people who inject drugs [42]. Future studies should aim to investigate the efficacy of policies that prevent eviction on stimulant use outcomes.

This study has a number of strengths, including the use of prospective cohort data allowing for the evaluation of changes in stimulant use patterns in relation to shifting contextual factors. Additionally, the study included a community-recruited sample of impoverished women, allowing for the focused evaluation of an under-studied population at significant risk for initiating or returning to stimulant use.

However, several limitations must also be considered in interpreting these results. First, while this study was prospective and longitudinal, because the assessments of independent and dependent variables were conducted concurrently at monthly time intervals, we cannot know for sure whether experiences of street or sheltered homelessness preceded stimulant use or vice versa. Second, data were obtained through participant self-report and may thus be susceptible to recall bias or underreporting of substance use behaviors due to social desirability. However, we would expect this to have biased results toward the null; thus, the actual effects would be expected to be at least as strong as those reported here. Third, this study took place in a single urban center, which may not be representative of the experiences of unstably housed women from other geographic localities. Fourth, as is true for any non-randomized study, potential unmeasured confounders—such as non-specific trauma [57,58]—may have influenced the associations found between the risk factors evaluated and return to stimulant use. Finally, the question on using marijuana as a substitute for other drugs was added late in the study, resulting in a lower sample size for this variable relative to others (Table 1). While there is no indication that individuals who were enrolled early had a differential risk of marijuana use, future studies with larger samples may help substantiate and clarify the early, non-significant findings reported here.

## 5. Conclusions

The current analysis demonstrates that, among impoverished women who do not use stimulants or who have recently abstained from stimulant use, experiencing street-homelessness is associated with an increased risk of initiating cocaine use while experiencing sheltered-homelessness or unstable housing is associated with methamphetamine use. Furthermore, experiences of homelessness and unstable housing among women are more strongly correlated with stimulant use initiation than other known predictors—including mental illness, race, income, and unmet subsistence needs. In an era marked by widespread polarization regarding causative factors and effective policy solutions related to the intersection of substance use and homelessness, these data suggest that increasing access to safe and permanent housing is not only critical to reducing stimulant use at the level of population health but is likely more important than access to mental healthcare and other social services alone.

## Figures and Tables

**Table 1:** Characteristics of homeless and unstably housed women (N = 245) in San Francisco (2016–2019).

	N (or Mean)	% (or SD)
Demographics		
Age (years), mean (SD)	51.6	(10.8)
Race/ethnicity		
White	64	(26.1%)
Black/African American	92	(37.6%)
Latina	37	(15.1%)
Multiracial	29	(11.8%)
Other	23	(9.4%)
Sexual orientation		
Heterosexual	181	(73.9%)
Homosexual/lesbian	16	(6.5%)
Bisexual	39	(15.9%)
None of the above	9	(3.7%)
Monthly income (dollars), mean (SD)	935.4	(662.1)
Housing		
Chose where to sleep based on avoiding violence	87	(35.5%)
Able to stay at the same place the next two weeks	212	(86.5%)
Slept at a homeless shelter in the past month	68	(27.8%)
Slept in a public place in the past month	56	(22.9%)
Mental Health Disorders and Treatment Adherence		
Mental health (depression, PTSD, or anxiety)		
No mental illness	61	(25.1%)
Mental illness; daily adherence to treatment	71	(29.2%)
Mental illness; less than daily adherence or untreated	111	(45.7%)
Depression (PHQ-9)		
Minimal to mild	125	(51.0%)
Moderate to severe	120	(49.0%)
PTSD score (PCL), mean (SD)	15.8	(5.8)
Anxiety score (GAD-9), mean (SD)	8.4	(5.6)
Cognitive function score (PROMIS-8), mean (SD)	24.3	(8.3)
Used marijuana as substitute for another substance ^1^	7	(29.2%)

^1^ Questions on drug substitution were introduced approximately 22 months after the study began enrollment and asked only among women who reported using marijuana at the current study visit (N = 24).

**Table 2:** Predictors of initiating cocaine use after a period of non-use among impoverished women, San Francisco, CA, USA (2016–2019).

	Adjusted Odds Ratio (AOR)	95% CI	*p*-Value
Age at V1 (per 10 units)	0.58 ^ǂ^	0.44, 0.78	<0.001 **
Race/ethnicity			
White	(ref)	--	--
Black/African American	0.47	0.22, 1.04	0.06
Latina	1.01	0.44, 2.33	0.98
Multiracial	0.93	0.33, 2.64	0.89
Other	0.54	0.16, 1.81	0.32
Income (per dollar)	1.00	1.00, 1.00	0.22
Unmet subsistence needs	1.58	0.87, 2.85	0.13
Chose where to sleep based on avoiding violence	1.22	0.65, 2.31	0.53
Ability to stay at same place the next two weeks	0.83	0.29, 2.34	0.72
Slept at a homeless shelter in the past month	1.07	0.51, 2.24	0.87
**Slept in a public place in the past month**	**2.10 ^ǂ^**	**1.04, 4.25**	0.038 **
Mental health (depression, PTSD, or anxiety)			
No mental illness	(ref)	--	--
Mental illness; daily adherence to treatment	0.92	0.44, 1.94	0.83
Mental illness; less than daily adherence or untreated	0.94	0.44, 2.00	0.87
Depression (PHQ-9)			
Minimal to mild	(ref)	--	--
Moderate to severe	0.82	0.44, 1.53	0.54
PTSD score (PCL)	1.00	0.96, 1.05	0.86
Anxiety score (GAD-9)	1.01	0.95, 1.06	0.85
Cognitive function score (PROMIS-8)	1.00	0.96, 1.04	0.97
Marijuana used as substitute for another substance	0.3	0.03, 2.57	0.27

^ǂ^ Estimates adjusted for age and slept in a public place. ** *p* < 0.05.

**Table 3:** Predictors of initiating methamphetamine use after a period of non-use among impoverished women, San Francisco, CA, USA (2016–2019).

	Adjusted Odds Ratio (AOR)	95% CI	*p*-Value
Age at V1 (per 10 units)	1.11	0.85, 1.45	0.45
Race/ethnicity			
White	(ref)	--	--
Black/African American	1.34	0.68, 2.65	0.40
Latina	0.75	0.29, 1.93	0.55
Multiracial	0.34	0.08, 1.41	0.14
Other	0.40	0.10, 1.65	0.21
Income (per dollar)	1.00	1.00, 1.00	0.63
Unmet subsistence needs	0.93	0.47, 1.82	0.83
Chose where to sleep based on avoiding violence	1.07	0.59, 1.93	0.83
**Ability to stay at same place the next two weeks**	**0.36 ^ǂ^**	**0.17, 0.79**	0.01 **
**Slept at a homeless shelter in the past month**	**2.57 ^ǂ^**	**1.37, 4.79**	0.003 **
Slept in a public place in the past month	1.45	0.74, 2.84	0.28
Mental health (depression, PTSD, or anxiety)			
No mental illness	(ref)	--	--
Mental illness; daily adherence to treatment	1.58	0.71, 3.51	0.27
Mental illness; less than daily adherence or untreated	1.56	0.72, 3.37	0.26
Depression (PHQ-9)			
Minimal to mild	(ref)	--	--
Moderate to severe	1.53	0.86, 2.73	0.15
PTSD score (PCL)	1.02	0.97, 1.06	0.42
Anxiety score (GAD-9)	1.04	1.00, 1.09	0.06
Cognitive function score (PROMIS-8)	0.97	0.93, 1.01	0.09
Marijuana use as substitute for another substance	2.59	0.51, 13.24	0.25

^ǂ^ Estimates adjusted for ability to stay at the same place for the next two weeks and slept in a homeless shelter. ** *p* < 0.05.

## Data Availability

Due to the confidentiality of sensitive information from a small and potentially identifiable group, data are not publicly available.
